# Comparison of re-revision rate and radiological outcomes between Kerboull-type plate and metal mesh with impaction bone grafting for revision total hip arthroplasty

**DOI:** 10.1186/s12891-023-06240-0

**Published:** 2023-02-20

**Authors:** Hotaka Ishizu, Tomohiro Shimizu, Fumio Sasazawa, Daisuke Takahashi, Mohamad Alaa Terkawi, Kaname Takahashi, Yusuke Ohashi, Masahiro Kanayama, Norimasa Iwasaki

**Affiliations:** 1 Department of Orthopaedic Surgery, Faculty of Medicine and Graduate School of Medicine, Hokkaido University, Kita-15 Nishi-7, Kita-Ku, Sapporo, 060-8638 Japan; 2 Department of Orthopaedic Surgery, Hakodate Central General Hospital, Hakodate, Hon-cho 33-2, 040-8585 Japan

**Keywords:** Kerboull-type plate, Revision total hip arthroplasty, Metal mesh, Impaction bone grafting, Vertical defect height

## Abstract

**Background:**

This study compared the re-revision rate and radiographic outcomes of revision total hip arthroplasty (THA) using a Kerboull-type acetabular reinforcement device (KT plate) with bulk structural allograft and metal mesh with impaction bone grafting (IBG).

**Methods:**

Ninety-one hips of 81 patients underwent revision THA for American Academy of Orthopedic Surgeons (AAOS) classification type III defects from 2008 to 2018. Of these, seven hips of five patients and 15 hips of 13 patients were excluded due to insufficient follow-up information (< 24 months) and large bone defects with a vertical defect height ≥ 60 mm, respectively. The current study compared the survival and radiographic parameters of 45 hips of 41 patients using a KT plate (KT group) and 24 hips of 24 patients using a metal mesh with IBG (mesh group).

**Results:**

Eleven hips (24.4%) in the KT group and 1 hip (4.2%) in the mesh group exhibited radiological failure. Moreover, 8 hips in the KT group (17.0%) required a re-revision THA, while none of the patients in the mesh group required a re-revision. The survival rate with radiographic failure as the endpoint in the mesh group was significantly higher than that in the KT group (100% vs 86.7% at 1-year and 95.8% vs 80.0% at 5-years, respectively; *p* = 0.032). On multivariable analysis evaluating factors associated with radiographic failure, there were no significant associations with any radiographic measurement. Of the 11 hips with radiographic failure, 1 (11.1%), 3 (12.5%), and 7 (58.3%) hips were of Kawanabe classification stages 2, 3, and 4, respectively.

**Conclusions:**

The findings of this study suggest that revision THA using KT plates with bulk structure allografts could provide poorer clinical outcomes than revision THA using a metal mesh with IBG. Although revision THA using KT plates with bulk structural allografts could set the true hip center, there is no association between a high hip center and clinical outcomes. The relationship between the position of the KT plate and the host bone might be considered more carefully.

## Background

Aseptic loosening, breakage of the implants, and acetabular bone defects due to osteolysis are still important complications of total hip arthroplasty (THA). Various techniques have been used to augment acetabular bone defects, including acetabular reinforcement devices such as the Kerboull acetabular plate [1, 2], acetabular trabecular metal augments, and impaction bone grafting (IBG). To loosen acetabular bone defects, acetabular reconstruction using a Kerboull acetabular reinforcement device and a bulk structural allograft is an effective approach and has produced good outcomes [3, 4]. Iwase et al. showed that acetabular reconstruction using a metal mesh with IBG provides good outcomes for moderate acetabular bone defects [5]. IBG is a well-known method that can restore bone stock deficiency while placing the hip rotation center around the true acetabulum.

In some cases, a re-revision surgery may be required following aseptic loosening and breakage of implants post-operatively. The loss of acetabular bone, especially in the superior area (such as Paprosky type 3A and 3B [6]), has been reported to be associated with the longevity of revision THA using the Kerboull reinforcement device and a bulk allograft [3]. Some groups have reported that the cup survival rate of THA using a metal mesh with IBG is influenced by the amount of the bone defect, degree of the wall defect, cup abduction angle, and body mass index (BMI) [5, 7]. Previous reports have indicated that it is important to place the acetabular component in its original position, and that achieving the optimal positioning reduces the incidence of loosening and dislocation [8, 9]. However, for large bone defects such as AAOS class III, if the acetabular component is placed in the original position, the bone grafts may become larger, leading to poor results [3]. For AAOS class III, there is a lack of consensus on the optimal placement of the acetabular component, i.e., whether to place the acetabular component in the original position with a larger bone graft or to allow a high hip center with a smaller bone graft. A proper understanding of the trends and characteristics of each device may help reduce complications, including aseptic loosening, breakage of implants, and frequent dislocations. Although there have been many studies describing the effects of each device, an evaluation of the comparative efficacy of the devices for cases with larger bone defect is lacking.

The purpose of this study was to compare the re-revision rate and radiographic features of revision THA using a Kerboull-type acetabular reinforcement device (KT plate) [10] with a bulk structural allograft and a metal mesh with IBG for patients with American Academy of Orthopedic Surgeons (AAOS) type III acetabular bone defects [11]. We hypothesized that revision THA using a metal mesh with IBG would be better for patients with larger bone defects than using a KT plate.

## Patients and Methods

This retrospective study was conducted in accordance with the ethical standards of the Declaration of Helsinki and was approved by the Institutional Review Board (# 015–0205). Between 2008 and 2018, 205 hips of 180 patients underwent revision THA at the two participating hospitals. The acetabular bone defects were classified according to the AAOS classification [11], and of the 205 hips of 180 patients, 184 hips of 162 patients had AAOS classification type III (combined cavitary and segmental bone loss).

**Fig. 1:**
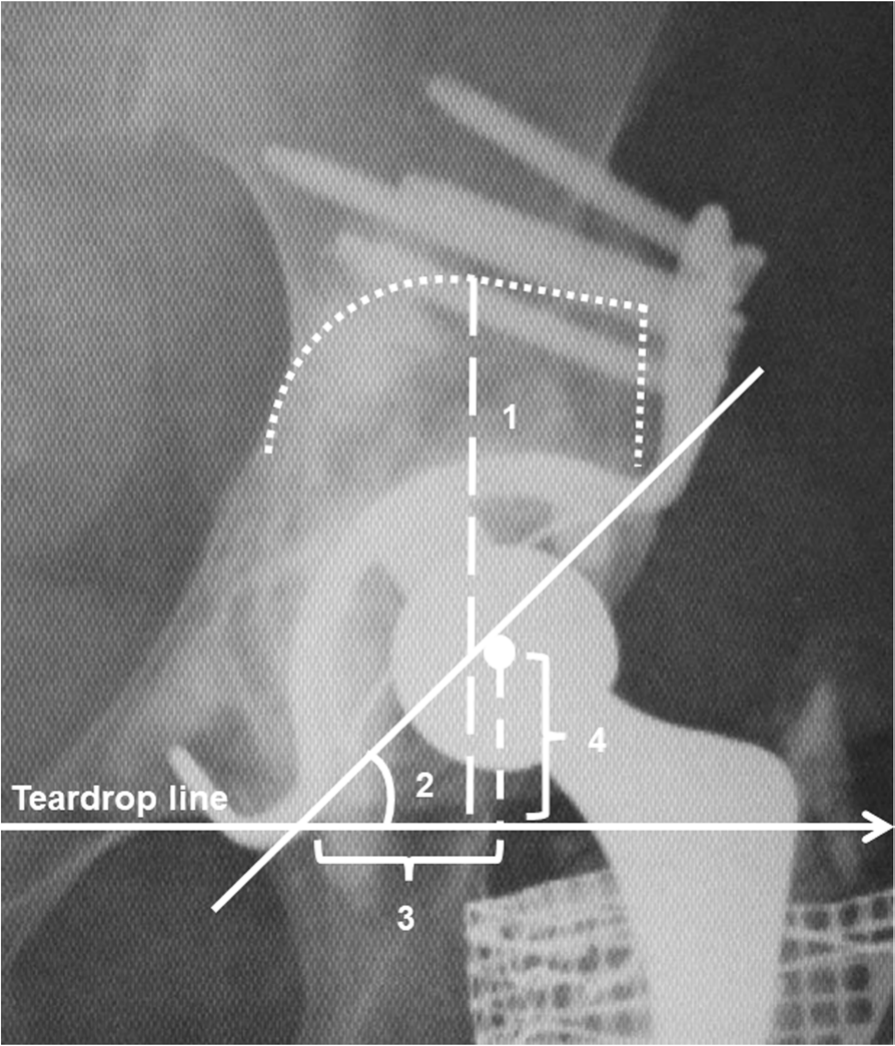
Anteroposterior (AP) radiographs of the bilateral hip joints were measured at each time point. Four parameters were measured and reported: 1) vertical defect height, 2) cup abduction angle, 3) horizontal migration, and 4) vertical migration

For AAOS type III acetabular bone defects, a KT plate was often used (Kyocera, Kyoto, Japan) combined with a bulk structural allograft or metal mesh with IBG. For implant selection, the vertical defect height, which was defined as the distance between the inter-teardrop reference line and the top of the acetabular bone defect, was measured using the preoperative radiographs (Fig. 1). Our group usually uses a bone graft of < 25 mm based on the findings in a previous report [7]. Considering a cup with a diameter varying from 44 to 48 mm and set at a 45-degree abduction angle, a vertical defect height of < 60 mm would allow the cup to set at the true hip center using a KT plate for placement at the original position (440,005 or 480,005-purity titanium, Kyocera, Kyoto, Japan) [12] or a metal mesh with IBG. If the vertical defect height was > 60 mm, we used a KT plate for high placement. The implant was selected by the surgeon based on the form of bone defect and the presumed outcomes and morbidity. The choice was made with the consent of the patient.

**Fig. 2:**
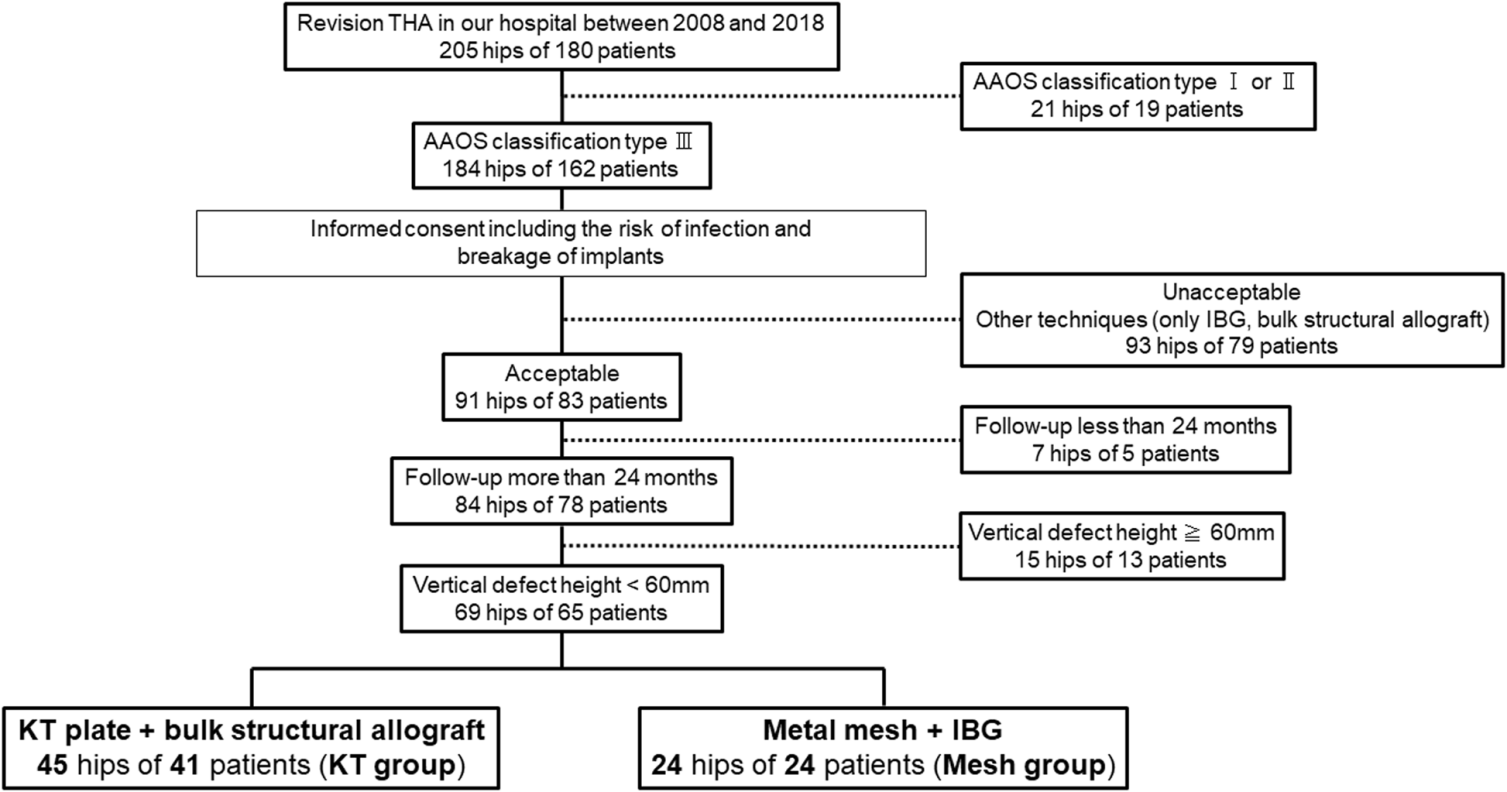
Study design

Acetabular reconstruction was performed for 91 hips of 83 patients after obtaining consent. Of these, 7 hips of 5 patients dropped out within 24 months for no discernible reason. Using the preoperative radiographs, we measured the vertical defect height, which was defined as the distance between the inter-teardrop reference line and the top of the acetabular bone defect. We excluded 15 hips from 13 patients who had a vertical defect height of ≥ 60 mm. Ultimately, we analyzed a total of 69 hips from 65 patients, of which 45 hips from 41 patients were in the KT group (KT plate with a bulk structural allograft) and 24 hips from 24 patients were in the mesh group (metal mesh with IBG) (Fig. 2).

Four surgeons practicing at the two participating hospitals performed the surgeries. All procedures were performed using the posterolateral approach, with the patient in the lateral position. We exposed the acetabulum and removed the loosening acetabular component, cement, and granulation tissue. Subsequently, we assessed the acetabular bone defects and identified the obturator foramen. In the KT group, the hook of the KT plate was placed under the obturator foramen and anatomical placement of the true hip center was performed. The extent and location of acetabular bone defects were assessed in detail. Based on this assessment, the allograft bone was molded and implanted into the defects as a structural bulk bone. The KT plate was fixed using at least two screws (range, 2–4) to obtain sufficient stability. In the mesh group, after the bone defects were contained using a metal mesh (X-Change, Stryker, Kalamazoo, Michigan, USA) using at least four cortical screws (range, 4–10), the morselized cancellous allograft bone chips were tightly impacted into the acetabular cavity containing hemispherical impactors and a metal hammer. The osteosclerotic femoral heads derived from osteoarthritis were used for all bone grafts. After these reconstructions, a cup of highly cross-linked polyethylene (X3 RimFit, Stryker) was cemented using standard bone cement (Simplex P, Stryker). For postoperative rehabilitation, patients used a walking frame and performed toe-touch weight-bearing activity, and partial weight bearing was allowed after 2 to 6 weeks. Subsequently, progressive weight-bearing was allowed as tolerated. All patients were permitted full weight bearing after 8 weeks postoperatively.

We evaluated the Japanese Orthopaedic Association (JOA) hip score, which consists of four subcategories: pain, range of motion, ability to walk, and activities of daily life [13]. We analyzed anteroposterior (AP) radiographs of the bilateral hip joints immediately postoperatively and at 1, 3, and 6 months; 1 year; and annually thereafter. We measured four parameters: 1) vertical defect height; 2) angle of inclination of the cup and teardrop line (cup abduction angle); 3) horizontal migration, defined as the distance between a perpendicular reference line drawn through the teardrop and the center of the femoral head; and 4) vertical migration, defined as the distance between the inter-teardrop reference line and the center of the femoral head [1, 14] (Fig. 1). In the KT-group, we evaluated the plate placement according to the Kawanabe classification [1]. Substantial migration was defined as a change in the cup abduction angle of more than 3° or cup migration of more than 3 mm. Radiological failure was defined by the following criteria: 1) substantial migration, and 2) breakage of the screws or device without change in inclination or migration [1, 14]. To search for more detailed causes of radiographic failure, we performed univariable and multivariable analyses for all the participants.

Comparisons between measurements were performed using Student’s t-test and chi-square tests. A *p*-value < 0.05 was considered statistically significant. Kaplan–Meier analysis was performed, with radiographic failure or a re-revision surgery as the endpoint. Multivariable analyses were conducted on all the participants and adjusted for age and BMI using a logistic regression model. Radiographic parameters were also included in the analysis. The data were analyzed using JMP Pro 14 software (SAS Institute, Japan).

## Results

Table 1 summarizes patient demographics at the time of surgery. The mean age and BMI of the KT group were significantly higher than those of the mesh group (*p* = 0.008 and *p* = 0.012, respectively). The male-to-female ratio and mean age were similar between the two groups (*p* = 0.938). In the KT group, eight hips demonstrated radiographic failure and three hips received re-revision surgery within 24 months. In contrast, no cases showed radiographic failure or received re-revision surgery within 24 months in the mesh group. The mean duration of the clinical follow-up period was 55.6 months (0–130) in the KT group and 75.4 months (25–149) in the mesh group (*p* = 0.022). The causes of revision surgery in the KT group were aseptic loosening in 43 hips, periprosthetic fracture in 1 hip, infection in 1 hip; and in the mesh group aseptic loosening in 20 hips, implant breakage in 1 hip, and infection in 3 hips (*p* = 0.138) (Table 1). The JOA hip score at the final follow-up was 81.3 points (standard deviation (SD): 10.8, range 62–97 points) in the mesh group, which was significantly higher than 69.9 points (SD: 12.1, range 32–91 points) in the KT group (*p* = 0.002).

**Table 1:** Clinical characteristics of the participants

	KT group (*N* = 45)	mesh group (*N* = 24)	*P-*value
Age [years]	73.2 (55–88)	67.0 (52–82)	**0.008**
Sex, male: female	4:41	2:22	0.938
BMI [kg/m^2^]	24.9 (15.2–39.1)	21.9 (16.9–29.4)	**0.012**
Diagnosis, cases			0.138
Aseptic loosening	43	20	
Implant breakage	0	1	
Periprosthetic fracture	1	0	
Infection	1	3	
Follow-up period [month]	55.6 (0–130)	75.4 (25–149)	**0.022**

Data presented as mean (range). BMI: Body mass index

The mean vertical defect height was 43.7 mm (SD: 7.7, range 25.5–57.2) in the KT group and 40.8 mm (SD: 11.0, range 21.7–59.9) in the mesh group (*p* = 0.206) (Table 2). The mean vertical migration was 17.2 mm (SD: 4.7, range 5.7–25.2) in the KT group and 24.1 mm (SD: 7.0, range 14.7–39.4) in the mesh group (*p* < 0.001). The mean horizontal migration was 29.0 mm (SD: 3.2, range 14.0–34.2) in the KT group and 30.6 mm (SD: 3.8, range 24.5–38.2) in the mesh group (*p* = 0.072). The mean cup abduction angle was 36.8° (SD: 8.1, range 10.2–51.0) in the KT group and 36.5° (SD: 8.0, range 20.6–53.0) in the mesh group (*p* = 0.882). In the KT group, 9, 24, and 12 hips were of Kawanabe classification stages 2, 3, and 4, respectively.

**Table 2:** The mean value for each survey item and the cause of reoperation in the KT group and the mesh group

	KT group	mesh group	*P*-value
Operation time, minutes	229 ± 66	186 ± 38	**0.005**
Blood loss, ml	441 ± 219	468 ± 210	0.636
Vertical defect height, mm	43.7 ± 7.7	40.8 ± 11.0	0.206
Vertical migration, mm	17.2 ± 4.7	24.1 ± 7.0	** < 0.001**
Horizontal migration, mm	29.0 ± 3.2	30.6 ± 3.8	0.072
Cup abduction angle, degree	36.8 ± 8.1	36.5 ± 8.0	0.882
Kawanabe classification			
Stage 2	9		
Stage 3	24		
Stage 4	12		
Second revision surgery, cases	8	0	**0.028**
Radiographic failure, cases	11	1	**0.035**
Causes, cases			0.834
Implant failure	8	1	
Periprosthetic fracture	1	0	
Infection	2	0	

Data presented as mean ± standard deviation

**Fig. 3:**
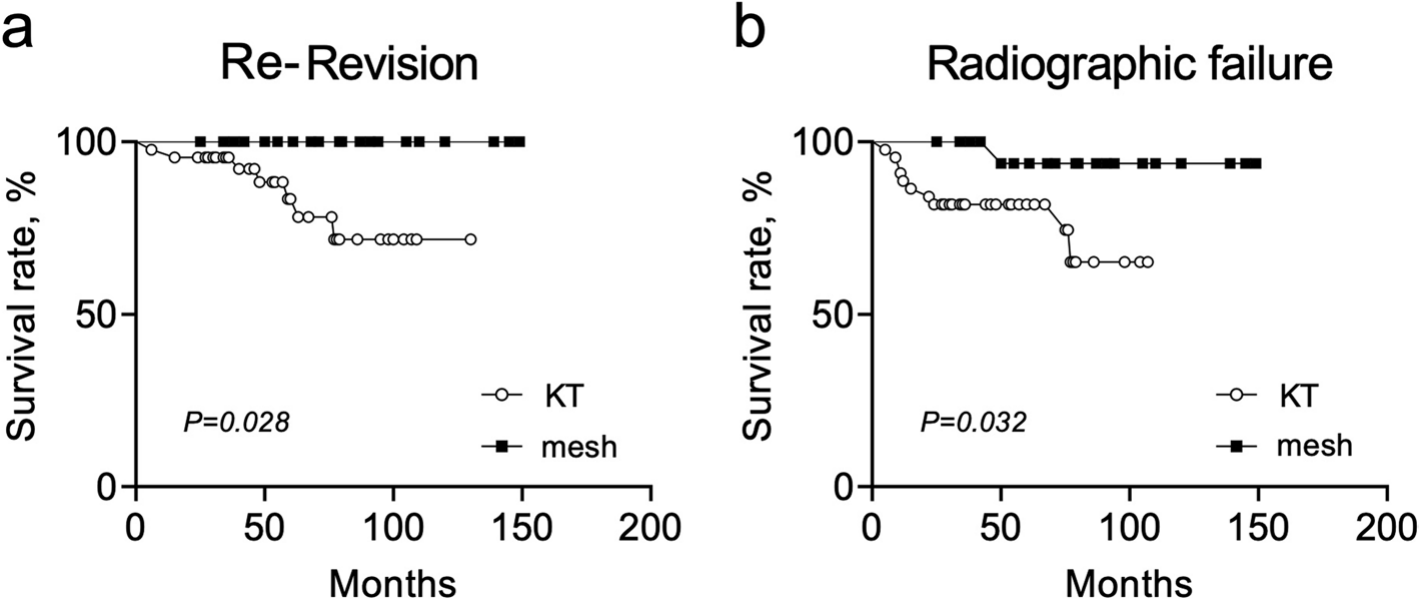
Kaplan–Meier survival analysis. **a** Kaplan–Meier curves showing the survival rate with second revision surgery for any reason as the endpoint. **b** Kaplan–Meier curves showing the survival rate with radiographic failure as the endpoint

Eight hips required re-revision surgery in the KT group, while no hips required re-revision surgery in the mesh group (17.8% [8/45] vs. 0.0% [0/24]) (*p* = 0.028). Eleven hips showed radiographic failure in the KT group, while only one showed failure in the mesh group (24.4% [11/45] vs. 4.2% [1/24]) (*p* = 0.035). The failures were not caused by the same surgeon. The causes of radiographic failure were as follows: implant failure (8 hips), infection (2 hips), and periprosthetic fracture of the acetabulum (1 hip) in the KT group, and screw or device breakage (1 hip) in the mesh group (*p* = 0.834). Of the 11 hips with radiographic failure, 1 (11.1%), 3 (12.5%), and 7 (58.3%) hips were of Kawanabe classification stages 2, 3, and 4, respectively. Kaplan–Meier analysis showed that the survival rate with re-revision surgery as the endpoint in the mesh group was significantly higher than that in the KT group (*p* = 0.028) (Fig. 3a). The survival rate with radiographic failure as the endpoint in the mesh group was significantly higher than that in the KT group (100% vs 86.7% at 1-year and 95.8% vs 80.0% at 5-years, respectively; *p* = 0.032) (Fig. 3b). No cases of dislocation occurred during the follow-up period in either group.

Univariate and multivariate logistic regression analyses of all the participants were performed to calculate the odds ratios (ORs) for the radiographic failure (Table 3). In the univariable and multivariable analysis, the surgical procedure (KT plate) had the highest OR (OR: 7.441, 95% Confidence Interval (CI): 0.898–61.647; *p* = 0.063, OR: 3.341, 95%CI: 0.235–47.400; *p* = 0.373, respectively).

**Table 3:** Odds ratios for the radiographic failure rates in all the participants

	Odds ratio	95% CI		*P*-value
Univariate analysis				
Age, years old	1.041	0.969	1.118	0.269
BMI, kg/m^2^	1.061	0.934	1.206	0.362
Operation procedure (KT plate)	7.441	0.898	61.647	0.063
Vertical defect height, mm	1.041	0.967	1.121	0.287
Vertical migration, mm	0.934	0.835	1.044	0.230
Horizontal migration, mm	0.906	0.760	1.080	0.271
Cup abduction angle, degree	0.939	0.871	1.013	0.102
Multivariate analysis				
Age, years old	1.012	0.934	1.098	0.760
BMI, kg/m^2^	1.059	0.903	1.242	0.483
Operation procedure (KT plate)	3.341	0.235	47.400	0.346
Vertical defect height, mm	1.091	0.959	1.241	0.162
Vertical migration, mm	0.933	0.768	1.132	0.470
Horizontal migration, mm	0.935	0.740	1.183	0.576
Cup abduction angle, degree	0.940	0.859	1.028	0.166

*CI* Confidential interval, *BMI* Body mass index

## Discussion

This study aimed to compare the clinical and radiological outcomes of revision cases of AAOS class III bone defects using a metal mesh with IBG and KT plates with bulk structural grafts. None of the patients in the mesh group required a re-revision surgery and only one patient experienced radiographic failure, suggesting that revision THA using a metal mesh with IBG could be a useful surgical option for patients with AAOS class III bone defects. In contrast, while the KT group achieved more optimal installation in terms of hip center, a higher failure rate was observed in this group compared to that in the mesh group.

Previous reports have indicated that it is important to place the acetabular component in its original position. Morag et al. revealed a significant correlation between cup height and functional outcomes, with better outcomes and survivorship noted with a cup placement < 35 mm proximal to the inter-teardrop line [15]. Conversely, some reports have reported good results for revision THA with a high hip center to a certain extent [16, 17]. Baba et al. suggested that a high hip center is effective in reducing bone graft volume [12]. Considering the good clinical and radiographic outcomes of the mesh group, tolerance of a high hip center with a smaller bone graft may be a suitable surgical strategy for patients with AAOS class III bone defects.

The clinical and radiographic results of the current study for patients using KT plates with bulk structural allografts were in contrast to those of previous studies [3, 4, 18]. In this study, Kawanabe stage 4 cases exhibited a higher ratio of radiographic failure. Many cases of Kawanabe classification stage 4, in which contact with the host bone was not obtained, resulted in implant failure due to the collapse of the bone graft and abduction of the KT plate. Although our group attempted to place the KT plate in the original position to achieve a true hip center when using a bone graft of < 25 mm, the position of the KT plate in relation to the host bone should be considered from a biomechanical standpoint [19]. In contrast, recent reports concluded that there is no difference between Kawanabe stage 3 and 4 [20, 21]; therefore, it was necessary to consider other reasons for our failure rate. Because Japanese people are more likely to have a smaller femoral head [12], it may have been more challenging to mold the bone graft. However, in this study, a quantitative assessment of the strength of the bone graft was not investigated. Additionally, Hooten et al. concluded that failure of structural acetabular allografts in revision surgery during the first 24 months was usually due to technical errors [22]. Therefore, we speculated that various factors, which are difficult to quantify, such as the poor morphology and fragility of the bone graft, and the skill of the surgeon, influenced the poor result of the KT plate.

This study had several limitations. The main limitation was that we could not investigate or compare the width of the bone graft because the measurement of the width of each device was performed using different standards [3, 5]. Therefore, the current study mainly evaluated vertical and horizontal migration and vertical defect height. Second, we did not compare patient satisfaction even though it has been shown that high hip center results in discrepancies in leg length, which may have a negative influence on the patient. These considerations are essential for determining whether a high hip center is optimal. Third, X-ray imaging was used exclusively for image evaluation in this study. Computed tomography images were not obtained; therefore, the three-dimensional bone defect size could not be evaluated. Fourth, the sample size of this study was relatively small, and it might not have sufficient power to detect statistically significant differences in radiological failure rates. When the power, alpha error, and effect size were set at 80%, 0.05, and 0.8, respectively, the statistically required sample size for radiological failure, as determined by this study, was 52 cases per group. Finally, various confounding factors such as the strength of the bulk bone and the skill of the operator were not quantified, and we could not rule out the possibility that these factors might have influenced the results.

In conclusion, the findings of this study suggest that revision THA using KT plates with bulk structure allografts could provide poorer clinical outcomes than revision THA using a metal mesh with IBG. Although revision THA using KT plates with bulk structural allografts could set the true hip center, there is no association between a high hip center and clinical outcomes. The relationship between the position of the KT plate and the host bone might be considered more carefully.

## Data Availability

All data supporting our findings are contained within the manuscript.

## Abbreviations

AAOS: American Academy of Orthopedic Surgeons
AP: Anteroposterior
BMI: Body mass index
IBG: Impaction bone grafting
JOA: Japanese Orthopaedic Association
OR: Odds ratio
THA: Total hip arthroplasty
